# Seroprevalence and current infections of canine vector-borne diseases in Nicaragua

**DOI:** 10.1186/s13071-018-3173-1

**Published:** 2018-11-12

**Authors:** Andrea Springer, Victor M. Montenegro, Sabine Schicht, Nikola Pantchev, Christina Strube

**Affiliations:** 1 Institute for Parasitology, Centre for Infection Medicine, University of Veterinary Medicine Hannover, Buenteweg 17, 30559 Hannover, Germany; 2 Laboratorio de Parasitología, Escuela de Medicina Veterinaria, Universidad Nacional, Campus Benjamín Núñez, Heredia, Costa Rica; 3 IDEXX Laboratories, Moerikestr. 28/3, 71636 Ludwigsburg, Germany

**Keywords:** Vector-borne diseases, Tick-borne diseases, Ticks, Zoonoses, *Rickettsia* spp., *Ehrlichia* spp., *Anaplasma* spp., Central America

## Abstract

**Background:**

Vector-borne diseases constitute a major problem for veterinary and public health, especially in tropical regions like Central America. Domestic dogs may be infected with several vector-borne pathogens of zoonotic relevance, which may also severely compromise canine health.

**Methods:**

To assess the prevalence of canine vector-borne diseases in Nicaragua, 329 dogs from seven cities, which were presented to the veterinarian for various reasons, were included in this study. Dogs were examined clinically and diagnostic blood samples were taken for analysis of packed cell volume (PCV) and presence of microfilariae as well as antigen of *Dirofilaria immitis* and antibodies to *Ehrlichia* spp., *Anaplasma* spp. and *Borrelia burgdorferi* (*sensu lato*) by use of a commercially available rapid ELISA. To detect current infections, specific PCRs for the detection of *E. canis*, *A. platys* and *A. phagocytophilum* were carried out on blood samples of the respective seropositive dogs. Microfilaremic blood samples, as well as *D. immitis* antigen positive samples were further subjected to PCR and subsequent sequencing for filarial species identification.

**Results:**

Antibodies against *Ehrlichia* spp. were present in 62.9% of dogs, while *Anaplasma* spp. seroprevalence was 28.6%. Antibodies against species of both genera were detected in 24.9% of dogs. *Borrelia burgdorferi* (*s*.*l*.) antibodies were not detected. *Dirofilaria immitis* antigen was present in six animals (1.8%), two of which also showed *D. immitis* microfilariae in buffy coat. In addition to *D. immitis*, *Acanthocheilonema reconditum* was identified by PCR and sequencing in two of four additional microfilaremic blood samples, which were tested negative for *D. immitis* antigen. Current *E. canis* infections as defined by DNA detection were present in 58.5% of *Ehrlichia*-seropositive dogs, while 5.3% of *Anaplasma*-seropositive dogs were PCR-positive for *A. platys*, 2.2% for *A. phagocytophilum* and 16.0% for both *Anaplasma* species. Current *E. canis* infection had a statistically significant negative impact on PCV, whereas no relationship between infection status and clinical signs of disease could be observed.

**Conclusions:**

These results indicate that canine vector-borne diseases are widespread in Nicaragua and that dogs may constitute a reservoir for human infection with *E. canis*, *A. phagocytophilum* and *D. immitis*. Thus, the use of repellents or acaricides to protect dogs from vector-borne diseases is strongly recommended.

## Background

Vector-borne pathogens constitute an important problem for veterinary and public health, especially in tropical regions where the climate is ideal for vectors such as ticks and mosquitoes [1]. Domestic dogs may be affected by several vector-borne diseases, including leishmaniosis, babesiosis, ehrlichiosis, anaplasmosis and canine heartworm disease, which may severely compromise canine health. Clinical signs are often unspecific, including fever, lymphadenopathy or weight loss with haematologic abnormalities including anaemia and thrombocytopenia [2]. Co-infections are common in endemic areas and may alter and/or potentiate clinical signs, complicating diagnosis and treatment [3]. However, dogs may also be infected without showing any signs of disease or haematologic abnormalities [4].

Many canine vector-borne diseases are of major zoonotic concern, including Lyme borreliosis, granulocytic anaplasmosis, ehrlichiosis and spotted-fever rickettsioses. Dogs may act as reservoirs and sentinels for human infection with these pathogens [4, 5]. In consequence, surveillance of canine vector-borne diseases may reveal infection risks for humans and potential disease emergence foci [2, 6]. For example, *Borrelia burgdorferi* seroprevalence in dogs in the USA was higher in areas with a large number of human Lyme borreliosis cases [7]. Similarly, dogs living in areas associated with human rickettsiosis outbreaks in Costa Rica showed a higher *Rickettsia* seroprevalence than dogs elsewhere [8].

For Central America, prevalence data on canine vector-borne diseases are relatively scarce. In recent years, surveys have been published for Costa Rica [9–11] and Panama [12, 13], demonstrating a high prevalence of *Ehrlichia* spp., followed by *Anaplasma* spp. infections. Furthermore, *Dirofilaria immitis* infections were detected in Costa Rica, with a high regional prevalence in provinces along the Pacific coast [9, 11]. Comparable prevalence rates for *Ehrlichia* spp., *Anaplasma* spp. and *D. immitis* were also detected in Mexico [14]. Regarding Nicaragua, which borders Costa Rica to the north, comparable large-scale studies are lacking. Only a small-scale study (*n* = 39 dogs) has been conducted so far [15].

Therefore, seroprevalence of antibodies to *Ehrlichia* spp., *Anaplasma* spp. and *B. burgdorferi* (*sensu lato*) as well as antigen of *D. immitis* in dogs from seven different localities in Nicaragua was assessed in this study. Seropositive dogs were further tested by pathogen-specific PCRs for current infections, and relationships with clinical signs were explored.

## Methods

### Clinical examination and sampling of dogs

**Fig. 1:**
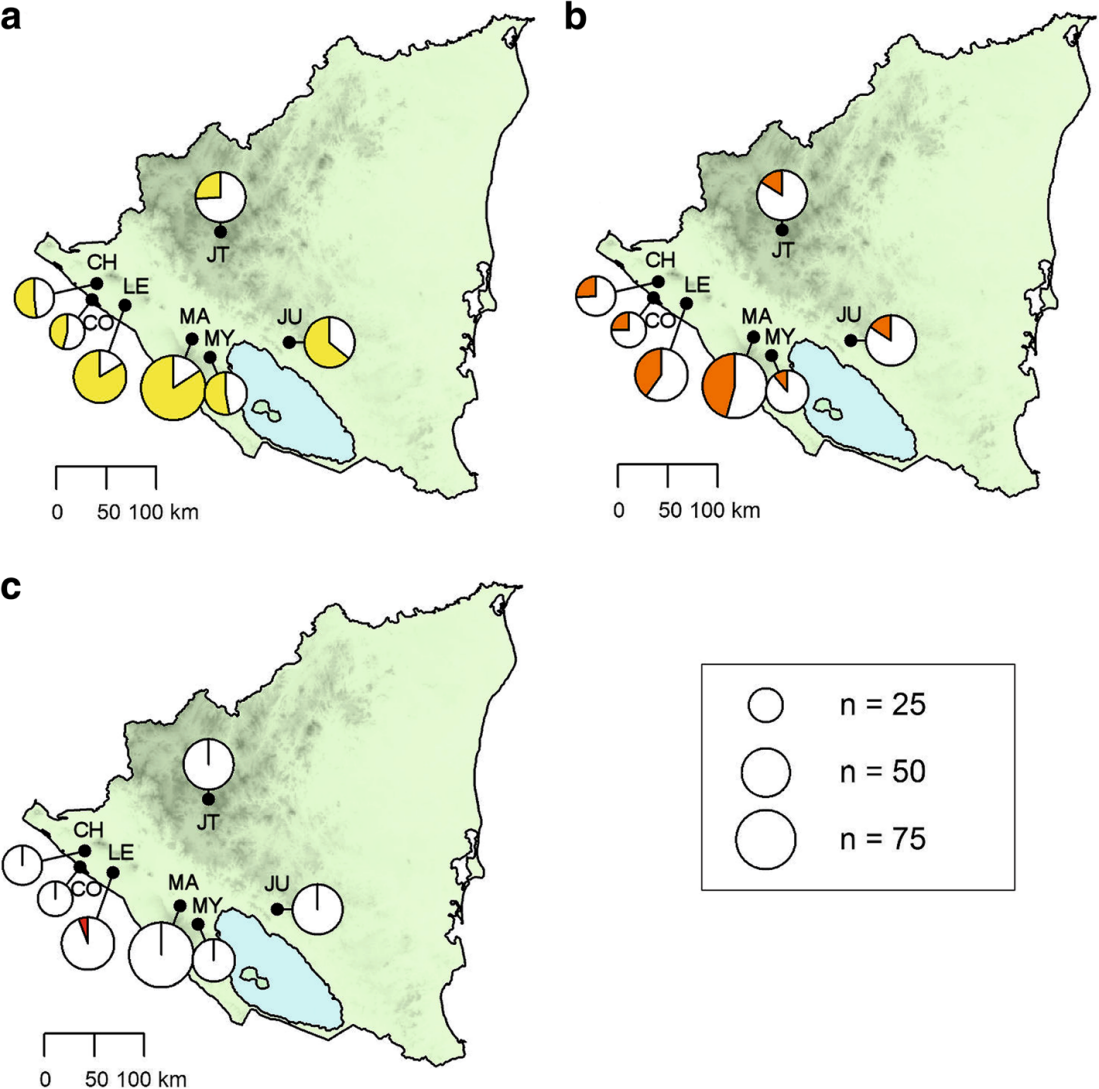
Seroprevalence of antibodies against *Ehrlichia* spp. (**a**) and *Anaplasma* spp. (**b**) as well as antigen of *Dirofilaria immitis* (**c**) in dogs tested by rapid ELISA in different cities of Nicaragua from September to December 2013. The size of pie charts corresponds to the number of dogs sampled at each site. *Abbreviations*: CH, Chinandega; CO, Corinto; JU, Juigalpa; JT, Jinotega; LE, Léon; MA, Managua; MY, Masaya

From September to October 2013, 329 dogs which were presented at veterinary clinics for various reasons were clinically examined and sampled in seven different cities in western Nicaragua. Four of these cities are located in the Pacific lowlands (Chinandega, León, Managua and Masaya), one at the Pacific coast (Corinto) and two in the central highlands (Jinotega and Juigalpa, Fig. 1). Per city, 31–83 dogs were sampled (Table 1). Most dogs were presented by their owners, whereas only in Managua, 12 dogs from an animal shelter were included in the study. Inclusion criteria for the dogs were the following: more than six months of age; not treated with ivermectin during the last six months nor with doxycycline during the last 12 months; and consent of the owner to use surplus samples for further examinations. Sex, age and breed of each dog were noted and a clinical examination was carried out. Diagnostic blood samples were taken from the cephalic or jugular vein and collected into serum and EDTA tubes. Packed cell volume (PCV) was determined by glass capillary centrifugation of EDTA blood. Remaining EDTA blood and serum was stored at -20 °C for further analyses.

**Table 1:** Number of dogs sampled, seroprevalence of *Anaplasma* spp. and *Ehrlichia* spp. and prevalence of *Dirofilaria immitis* antigen as determined by rapid ELISA, at the different sampling locations in Nicaragua

City	Geographical region	No. of dogs sampled	Seroprevalence of *Anaplasma* spp.	Seroprevalence of *Ehrlichia* spp.	Seroprevalence of *Borrelia burgdorferi* (*s*.*l*.)	Prevalence of *D. immitis*	Prevalence of tick infestation
Corinto	Pacific coast	24	6/24 (25.0%)	11/24 (45.8%)	0	0	5/24 (20.8%)
León	Pacific lowlands	55	22/55 (40.0%)	46/55 (83.6%)	0	6/55 (10.9%)	40/55 (72.7%)
Chinandega	Pacific lowlands	31	8/31 (25.8%)	16/31 (51.6%)	0	0	14/31 (45.2%)
Managua	Pacific lowlands	83	38/83 (45.8%)	70/83 (84.3%)	0	0	36/83 (43.4%)
Masaya	Pacific lowlands	36	4/36 (11.1%)	19/36 (52.8%)	0	0	14/36 (38.9%)
Jinotega	Central highlands	50	8/50 (16.0%)	13/50 (26.0%)	0	0	23/50 (46.0%)
Juigalpa	Central highlands	50	8/50 (16.0%)	32/50 (64.0%)	0	0	27/50 (54.0%)
Total		329	94/329 (28.6%)	207/329 (62.9%)	0/329 (0.0%)	6/329 (1.8%)	159/329 (48.3%)

### Screening of blood samples for vector-borne pathogens

Canine serum samples were tested for antibodies against *Anaplasma* spp., *Ehrlichia* spp. and *B. burgdorferis* (*s*.*l*.), as well as antigen of *D. immitis*, by use of a commercially available rapid ELISA (SNAP® 4Dx® Plus, IDEXX Laboratories Inc., Westbrook, ME, USA). According to Stillman et al. [16] the sensitivity and specificity of the test system are 93.2 and 99.2% for *A. phagocytophilum*, 89.2 and 99.2% for *A. platys*, 96.7 and 98.8% for *B. burgdorferi* (*s*.*l*.), 97.8 and 92.3% for *E. canis*, and 98.9 and 99.3% for *D. immitis*. Furthermore, a cross-reactivity of *E. canis* antigens with anti-*E. chaffeensis* antibodies was shown. Due to documented cross-reactivity between *A. phagocytophilum* and *A. platys* [17], as well as the reactivity to *E. canis*, *E. chaffeensis* and *E. ewingii* [16, 18], we refer to *Anaplasma* spp. and *Ehrlichia* spp. as results in the present study. Additionally, buffy coat of all dogs was investigated microscopically for presence of microfilariae. This technique has a comparable sensitivity to the Knott’s test, if at least 25 microfilariae are present per ml of blood sample [19].

To determine which *Anaplasma*- and *Ehrlichia*-seropositive dogs were currently infected (as defined by DNA detection) with *E. canis*, *A. phagocytophilum* and *A. platys*, respectively, species-specific PCRs were carried out. DNA was isolated from blood samples using the Nucleospin® 8 Blood Kit (Macherey-Nagel GmbH & Co. KG, Düren, Germany) according to the manufacturer’s instructions.

Samples were tested for *A. phagocytophilum* by employing a nested PCR targeting a 546 bp fragment of the *16S* rRNA gene. Primers used were ge3a and ge10r in a first and ge9f and ge2 in a second PCR round [20]. In each round, the 12.5 μl reaction volume contained 6.25 μl DreamTaq® PCR Mastermix (Thermo Fisher Scientific Inc., Waltham, MA, USA), 0.5 μl of each primer (10 μM each), 4.25 μl deionized water and 1 μl template DNA. In the second round, template DNA was represented by the PCR product of the first round. The thermoprofile was the same for both rounds and consisted of an initial denaturation step at 95 °C for 2 min, followed by 40 cycles at 95 °C for 30 s, 55 °C for 30 s and 72 °C for 1 min, and a final elongation step at 72 °C for 5 min.

To detect *A. platys*, a nested PCR targeting a 678 bp fragment of the *16S* rRNA gene was carried out using primer sets 8F and 1448R for a first and EHR16SR and PLATYS for a second PCR round [21]. Oligonucleotide concentration and reaction set-up were as described above except that 2.5 μl template DNA was used and the amount of water was adjusted accordingly. The thermoprofile of the first round consisted of an initial denaturation at 95 °C for 2 min, followed by 40 cycles at 95 °C for 1 min, 45 °C for 1 min and 72 °C for 2 min, and final extension at 72 °C for 5 min. The thermoprofile of the second round consisted of an initial denaturation at 95 °C for 1 min, followed by 40 cycles at 95 °C for 30 s, 55 °C for 30 s and 72 °C for 30 s, and a final extension at 72 °C for 5 min.

For detection of *E. canis*, a 389 bp fragment of the *16S* rRNA gene was targeted by nested PCR using primer pairs ECC and ECB in a first and ECAN5 and HE3 in a second PCR round [22, 23]. In the first round, the 25 μl reaction volume contained 12.5 μl DreamTaq® PCR Mastermix, 0.5 μl of ECC (0.4 μM), 0.5 μl of ECB (10 μM), 9.5 μl deionized water and 2 μl template DNA, while in the second round, 0.5 μl each of primers ECAN5 (0.4 μM) and HE3 (10 μM) as well as 2.5 μl of the PCR-product of the first round were used, and the amount of water was adjusted to 9 μl. The thermoprofile consisted of an initial denaturation at 95 °C for 2 min, followed by 40 cycles at 95 °C for 1 min, 60 °C for 1 min and 72 °C for 1 min, and a final extension at 72 °C for 5 min in the first round. In the second round, a thermoprofile of initial denaturation at 95°C for 1 min, followed by 40 cycles at 95 °C for 30 s, 55 °C for 30 s and 72 °C for 30 s, and a final extension at 72 °C for 5 min was used. In all PCR set-ups, positive and no template controls were included.

Samples which were positive for *D. immitis* antigen in the ELISA or contained microfilariae in buffy coat were subjected to a PCR targeting the internal transcribed spacer (ITS) 1-5.8S rDNA-ITS2 complex by use of primers NC2 and NC5 [24]. The 50 μl reaction volume contained 1 μl *Taq* polymerase (5 PRIME GmbH, Hilden, Germany), 5 μl 10× buffer, 2 μl of each primer (10 μM each), 1 μl deoxynucleotide triphosphates (10 mM each), 37 μl deionized water and 2 μl template DNA. The thermoprofile consisted of an initial denaturation step at 95 °C for 3 min, 30 cycles at 95 °C for 30 s, 55 °C for 30 s, 72 °C for 30 s, followed by final elongation at 72 °C for 10 min. After visualization on 1% agarose gels, bands of the expected size between 1000 and 1500 bp were ligated into a pCR™4-TOPO® TA vector and cloned into One Shot Top10 chemically competent *Escherichia coli* (TOPO® TA Cloning kit, Thermo Fisher Scientific GmbH, Dreieich, Germany). After plasmid extraction and purification (NucleoSpin Plasmid kit, Macherey-Nagel GmbH & Co. KG, Düren, Germany), the insert was sequenced at the Seqlab Sequence Laboratories (Göttingen, Germany).

### Statistical analyses

Statistical analyses were conducted in R v. 3.3.1 [25]. To investigate which factors influenced the probability of *Anaplasma* spp. and *Ehrlichia* spp. seropositivity in the rapid ELISA, generalized linear models (GLMs) with binomial error structure and logit-link were constructed (R function ‘glm’). Animal age, sex, breed (dichotomized into “with breed”/“mongrel”), city of sampling and tick infestation at the time of examination were included as predictors. Furthermore, *Anaplasma* spp. infection was included as a predictor in the model for *Ehrlichia* spp., and vice-versa, to examine associations between seroprevalence for these pathogens. A third model was constructed to examine which factors influenced the likelihood of a positive result for one or more pathogens in the rapid ELISA in general. Full models were compared to null models containing only an intercept term in a likelihood ratio test (R function ‘anova’, test = ‘chisq’). Model fit was assessed by Hosmer-Lemeshow-tests and inspection of Receiver Operating Characteristic (ROC) curves. Multiple comparisons (Tukey contrasts) between all levels of the factor “city” were performed using the function ‘glht’ (package *multcomp* [26]), with single-step *P*-value adjustment.

The influence of current infections with *A. platys*, *A. phagocytophilum* and *E. canis* (PCR results) on packed cell volume (PCV) was investigated using a linear mixed model (LMM, package *lme4* [27]). Because animal age and sex may affect PCV in dogs [28], these variables were included as additional fixed factors, while the city of sampling was included as a random factor. Because *A. platys* and *A. phagocytophilum* infections were highly correlated, these factors were investigated in separate models. Full LMMs were compared to null models containing only the random factor in a likelihood ratio test (R function ‘anova’, test = ‘chisq’). To validate LMM assumptions, normality and homogeneity of model residuals were investigated graphically.

The proportion of anorectic dogs was compared between *E. canis*-, *A. platys*- and *A. phagocytophilum*-infected and non-infected dogs using Fisher’s exact test.

Since only six dogs were positive for *D. immitis* antigen, these infections were not considered in statistical analyses.

## Results

### Clinical presentation of dogs

During clinical examinations, tick infestation was noted on 159 of the 329 dogs (48.3%), while flea and lice infestation were noted on 63 (19.1%) and 9 (2.7%) dogs, respectively. Pale mucous membranes were apparent in 129 dogs (39.2%), while anorexia was reported for 19 dogs (5.8%). Two dogs (0.6%) showed apathy, fever, epistaxis and abdominal pain. Lymphadenopathy was noted in one dog (0.3%). Other common clinical anomalies included nail overgrowth (*n* = 50; 15.2%), alopecia (*n* = 31; 9.4%), cough (*n* = 9; 2.7%) and eye discharge (*n* = 8; 2.4%).

### Seroprevalence of Rickettsiales and *D*. *immitis*

Antibodies against *Ehrlichia* spp. were detected in 62.9% (207/329) of dogs, while *Anaplasma* spp. seroprevalence was 28.6% (94/329). Antibodies against both *Ehrlichia* and *Anaplasma* spp. were detected in 82 animals (24.9%), demonstrating exposure to species of both genera. Antibodies against *B. burgdorferi* (*s*.*l*.) were not detected. Regional seroprevalences are presented in Table 1 and Fig. 1.

Statistical analyses revealed a significant association of *Ehrlichia* and *Anaplasma* seropositivity; animals that were seropositive for *Ehrlichia* spp. had approximately 5 times higher odds of testing positive for *Anaplasma* antibodies as well, and vice versa (GLM, Table 2, models A and B). Additionally, a significant effect of age on the likelihood of *Ehrlichia* seropositivity was observed, as well as on the likelihood of a positive ELISA result in general (GLM, Table 2, model C), with increasing probability in older dogs. Furthermore, significant differences were observed between the different sampling locations. Particularly, the odds of testing seropositive for *Ehrlichia* spp. were significantly lower in the city of Jinotega compared to Managua, Juigalpa and Léon, whereas for *Anaplasma* spp., a significant difference was only observed between Managua and Juigalpa, with a higher risk in Managua. Considering the results for *Anaplasma* spp., *Ehrlichia* spp. and *D. immitis* together, the odds of testing seropositive for any of these pathogens were significantly higher in Managua and Léon compared to Jinotega, in Managua compared to Corinto and Masaya, and in Masaya compared to Léon (Table 2). In contrast, animal sex and breed (with breed/mongrel) had no influence on seropositivity. Furthermore, tick infestation at the time of presentation was also not associated with seropositivity.

**Table 2:** Results of binomial GLMs testing the influence of different predictor variables on the probability of *Ehrlichia* spp. (Model A) and *Anaplasma* spp. (Model B) seropositivity and for a positive result in the rapid ELISA in general (Model C), amongst 329 dogs from Nicaragua

	Model A: *Ehrlichia* spp. seropositive					Model B: *Anaplasma* spp. seropositive					Model C: SNAP® 4Dx® Plus positive				
	Estimate	SE	*Z*	*P*	OR	Estimate	SE	*Z*	*P*	OR	Estimate	SE	*Z*	*P*	OR
Intercept	-1.85	0.44	-4.26	**<0.001**		-2.25	0.49	-4.55	**<0.001**		-1.13	0.39	-2.91	**0.004**	
Sex (ref. male)	0.09	0.27	0.34	0.735	1.1	0.09	0.27	0.32	0.751	1.09	0.04	0.26	0.15	0.883	1.04
Age	0.11	0.05	2.33	**0.020**	1.11	-0.02	0.05	-0.44	0.663	0.98	0.13	0.05	2.69	**0.007**	1.14
Breed (ref. “with breed”)	-0.29	0.29	-0.99	0.321	0.75	-0.13	0.29	-0.43	0.669	0.88	-0.08	0.29	-0.28	0.779	0.92
*Anaplasma* spp. seropositive	1.61	0.37	4.40	**<0.001**	5	–	–	–	–	–	–	–	–	–	–
*Ehrlichia* spp. seropositive	–	–	–	–	–	1.62	0.37	4.42	**<0.001**	5.03	–	–	–	–	–
Tick infestation	0.18	0.28	0.65	0.513	1.2	0.05	0.28	0.16	0.871	1.05	0.26	0.27	0.96	0.335	1.3
City^a^															
JU - JT	1.90	0.48	3.93	**0.002**	6.66	-0.59	0.59	-0.99	0.953	0.56	1.28	0.44	2.89	0.058	3.61
MA - JT	2.42	0.47	5.09	**<0.001**	11.21	0.79	0.50	1.56	0.694	2.2	2.36	0.46	5.16	**<0.001**	10.55
MY - JT	1.31	0.52	2.51	0.156	3.7	-0.79	0.71	-1.11	0.922	0.46	0.51	0.49	1.04	0.945	1.66
CO - JT	0.84	0.58	1.46	0.768	2.33	0.35	0.67	0.53	0.998	1.43	0.51	0.54	0.95	0.964	1.67
CH - JT	1.41	0.54	2.64	0.113	4.11	0.21	0.61	0.34	1.000	1.23	1.13	0.50	2.28	0.252	3.09
LE - JT	2.58	0.52	4.98	**<0.001**	13.14	0.46	0.53	0.88	0.975	1.59	2.13	0.48	4.42	**<0.001**	8.43
MA - JU	0.52	0.45	1.17	0.904	1.68	1.37	0.47	2.95	**0.048**	3.95	1.07	0.45	2.38	0.207	2.92
MY - JU	-0.59	0.48	-1.23	0.882	0.55	-0.20	0.68	-0.30	1.000	0.82	-0.78	0.47	-1.65	0.647	0.46
CO - JU	-1.05	0.55	-1.89	0.481	0.35	0.94	0.65	1.44	0.771	2.56	-0.77	0.53	-1.46	0.769	0.46
CH - JU	-0.48	0.50	-0.97	0.959	0.62	0.79	0.59	1.34	0.823	2.21	-0.15	0.48	-0.32	1.000	0.86
LE - JU	0.68	0.49	1.38	0.812	1.97	1.05	0.49	2.14	0.316	2.85	0.85	0.48	1.76	0.575	2.34
MY - MA	-1.11	0.47	-2.36	0.216	0.33	-1.57	0.59	-2.65	0.106	0.21	-1.85	0.48	-3.84	**<0.001**	0.16
CO - MA	-1.57	0.54	-2.91	0.055	0.21	-0.43	0.56	-0.77	0.987	0.65	-1.84	0.54	-3.43	**0.011**	0.16
CH - MA	-1.00	0.50	-2.00	0.413	0.37	-0.58	0.50	-1.16	0.903	0.56	-1.23	0.50	-2.45	0.178	0.29
LE - MA	0.16	0.50	0.32	1.000	1.17	-0.32	0.38	-0.84	0.979	0.73	-0.22	0.51	-0.44	0.999	0.8
CO - MY	-0.46	0.57	-0.82	0.983	0.63	1.14	0.74	1.54	0.712	3.13	0.01	0.55	0.01	1.000	1.0
CH - MY	0.11	0.54	0.20	1.000	1.11	0.99	0.70	1.41	0.785	2.7	0.62	0.52	1.20	0.893	1.86
LE - MY	1.27	0.54	2.36	0.211	3.55	1.25	0.63	1.98	0.418	3.49	1.62	0.53	3.09	**0.033**	5.08
CH - CO	0.57	0.60	0.95	0.964	1.77	-0.15	0.67	-0.22	1.000	0.86	0.62	0.57	1.08	0.933	1.85
LE - CO	1.73	0.60	2.89	0.059	5.65	0.11	0.61	0.18	1.000	1.11	1.62	0.58	2.79	0.077	5.05
LE - CH	1.16	0.55	2.12	0.336	3.2	0.26	0.53	0.49	0.999	1.29	1.00	0.53	1.88	0.489	2.73

Full models were significantly different from null models containing only an intercept: likelihood ratio test, *χ*^2^ = 92.3, *df* = 11, *P* < 0.001 (Model A); *χ*^2^ = 53.7, *df* = 11, *P* < 0.001 (Model B); and *χ*^2^ = 60.5, *df* = 11, *P* < 0.001 (Model C). Significant *P*-values (≤ 0.05) are printed in bold

^a^Multiple comparisons between levels of the factor “City” were performed using Tukey contrasts with single-step *P*-value adjustment.

*Abbreviations: JU*, Juigalpa; *JT*, Jinotega; *MA*, Managua; *MY*, Masaya; *CO*, Corinto; *CH*, Chinandega; *LE*, Léon; *OR*, odds ratio; *SE*, standard error

### Current infections and their clinical impact

In six dogs (1.8%), which were all sampled at the city of Léon, *D. immitis* antigen was detected. In two of these samples, microfilariae were microscopically observed and confirmed as *D. immitis* by PCR and sequencing. Microfilariae were also observed in four additional samples, which did not test positive for *D. immitis* antigen. In two of these samples, *Acanthocheilonema reconditum* was identified by PCR and sequencing.

Current *E. canis* infection, as determined by amplification of the pathogen’s DNA, was detected in 58.5% (121/207) of *Ehrlichia*-seropositive dogs, while *Anaplasma* DNA was detected in 23.4% (22/94) of seropositive samples. Among the 94 animals tested for *Anaplasma* DNA, five dogs (5.3%) were single-infected with *A. platys*, two dogs (2.2%) were single-infected with *A. phagocytophilum* and 15 dogs (16.0%) were co-infected with both species. Among the 85 animals tested by PCR for all three pathogens, four animals (4.7%) were co-infected with *E. canis* and *A. platys*, one animal (1.2%) with *E. canis* and *A. phagocytophilum* and eight animals (9.4%) carried all three pathogens (Table 3).

**Table 3:** Single and multiple infections among the 85 Nicaraguan dogs tested for *Anaplasma* and *Ehrlichia* DNA by PCR

No. of animals	Single infection			Multiple infection			
	Apl	Aph	Ec	Apl + Aph	Apl + Ec	Aph + Ec	Apl + Aph + Ec
85	0 (0.0%)	1 (1.2%)	41 (48.2%)	7 (8.2%)	4 (4.7%)	1 (1.2%)	8 (9.4%)

*Abbreviations*: Apl, *Anaplasma platys*; Aph, *Anaplasma phagocytophilum*; Ec, *Ehrlichia canis*

**Fig. 2:**
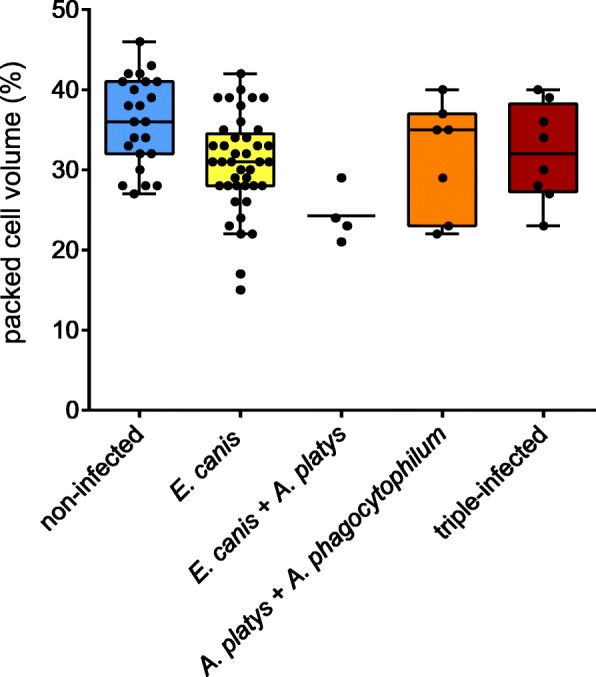
Packed cell volume of non-infected, mono-infected and co-infected dogs in the subset of animals tested by PCR for current infections (defined as DNA detection) with *Ehrlichia canis* and *Anaplasma* spp. (*n* = 85). Since only one dog each was mono-infected with *A. phagocytophilum* and co-infected with *E. canis* and *A. phagocytophilum*, respectively, these were not plotted. No mono-infections with *A. platys* were found in this data subset. Ends of the boxes define the 25th and 75th percentiles, with a line at the median and whiskers extending to 1.5 the interquartile range or up to the maximum/minimum value

In the clinical examination, 50.4% (61/121) of dogs with current *E. canis* infection, 75.0% (15/20) of dogs with current *A. platys* infections and 64.7% (11/17) of dogs with current *A. phagocytophilum* showed pale mucous membranes. However, 9 (45.0%) and 6 (35.3%) of these *A. platys*- and *A*. *phagocytophilum*-infected dogs were co-infected with *E. canis*, respectively. Figure 2 shows the distribution of PCV in non-infected, mono-infected and co-infected dogs in the subset of 85 animals which were tested by PCR for current infections with all three pathogens. In the linear mixed model, only current *E. canis* infection showed a statistically significant negative impact on PCV, while no significant effect of current *A. platys* or *A. phagocytophilum* infection on PCV was found (LMM, Table 4).

**Table 4:** Results of LMMs testing the influence of animal sex, age and current infections (as defined by DNA detection) with *Ehrlichia canis* and *Anaplasma platys* (Model A) as well as *A. phagocytophilum* (Model B) on packed cell volume (PVC) of 85 dogs from Nicaragua

	Estimate	SE	*df*	*t*	*P*
Model A					
Intercept	37.35	1.89	80	19.79	**<0.001**
Sex (ref. male)	0.15	1.31	80	0.11	0.909
Age	-0.33	0.26	80	-1.24	0.217
*E. canis* infection	-4.95	1.38	80	-3.58	**<0.001**
*A. platys* infection	-2.9	1.59	80	-1.83	0.072
Model B					
Intercept	36.75	2.02	80	18.19	**<0.001**
Sex (ref. male)	-0.07	1.33	80	-0.05	0.961
Age	-0.28	0.27	80	-1.03	0.306
*E. canis* infection	-4.95	1.43	80	-3.47	**<0.001**
*A. phagocytophilum* infection	-0.7	1.73	80	-0.4	0.688

Full models were significantly different from a null model containing only the random factor “City”: likelihood ratio test, *χ*^2^ = 15.28, *df* = 4, *P* = 0.004 (Model A); and *χ*^2^ = 11.98, *df* = 4, *P* = 0.017 (Model B). Significant *P*-values (≤ 0.05) are printed in bold

*Abbreviation: SE*, standard error

Twelve of 19 dogs for which anorexia was reported were PCR-positive for *E. canis*, with one individual also carrying current *A. platys* and *A. phagocytophilum* infections. However, for neither pathogen there was a significant difference in the occurrence of anorexia among infected and non-infected dogs (Fisher’s exact test, *P* = 0.1037, *P* = 1 and *P* = 1, respectively).

## Discussion

Dogs may be affected by several vector-borne diseases in the tropics, including important zoonoses. Here, detected seroprevalence rates among 329 dogs from Nicaragua for *Ehrlichia* spp. and *Anaplasma* spp. were considerably higher than those recently reported for Costa Rica (38.2% and 6.4%, respectively [9]) and Mexico (30.8% and 9.9%, respectively [14]). This could be due to the fact that dogs were only sampled in western parts of Nicaragua, characterized by less rainfall and a higher human population density than the hot and humid Caribbean lowlands in the eastern part of the country. In the abovementioned studies, a higher prevalence of canine vector-borne diseases has also been detected in western compared to central and eastern parts of Costa Rica and Mexico.

Furthermore, *Ehrlichia*- and *Anaplasma*-seropositivity were significantly associated with each other. In experimentally infected dogs it has been shown that concurrent *Ehrlichia*-infection intensifies the humoral immune response to *A. platys* and results in a more persistent *A. platys* infection [29], which may contribute to this phenomenon. Furthermore, both *Ehrlichia* and *A. platys* are predominantly transmitted by the brown dog tick *Rhipicephalus sanguineus* (*s*.*l*.), which was the most common tick species parasitizing the dogs in this study [30].

*Borrelia burgdorferi* (*s*.*l*.) antibodies were not detected, which is in line with the very low seroprevalences reported from Mexico and Costa Rica [9, 14], confirming the fact that Central America is not a Lyme borreliosis endemic region. The expected vector for *B. burgdoferi* (*s*.*l*.) are ticks of the genus *Ixodes*, which seem to be rather rare as parasites of domestic animals in Nicaragua [30, 31].

Several risk factors for seropositivity for tested vector-borne diseases were explored. A similar study conducted in Costa Rica reported a higher risk of seropositivity for mongrels compared to dogs of a certain breed as well as a significant effect of sex [9], which could not be confirmed in the current study. However, increasing age was identified as a risk factor for *Ehrlichia* spp. seropositivity as well as for a positive ELISA result in general, which most likely reflects cumulative pathogen exposure over the animals’ lifetime. Similarly, a significantly lower seropositivity rate of dogs under one year of age compared to older dogs was found in Mexico [14]. Furthermore, significant differences between sampling locations were found. High seroprevalences were found in the capital city, Managua, which differed significantly from the lower rates detected in Jinotega, Masaya and Corinto. However, a general pattern concerning the distribution of seropositivity, e.g. differences between coastal and highland locations, cannot yet be established. Furthermore, data on seroprevalences in the eastern parts of the country are still missing, and should be obtained in future studies.

*Dirofilaria immitis* antigen was detected in six dogs, which were all sampled in the city of Léon, near the Pacific coast. Similarly, *D. immitis* infections in Costa Rica were almost exclusively detected in districts bordering the Pacific coast [9, 11]. Higher *D. immitis* prevalence in coastal regions has also been observed in Mexico [32]. Additionally, microfilariae in two dogs were identified as *A. reconditum*. In neighbouring Costa Rica, *A. reconditum* infections have been detected in 11.6% of tested dogs, while 15% of dogs showed *D. immitis* infections [11]. In contrast to mosquito-borne dirofilariosis, *A. reconditum* is mainly transmitted by fleas and possibly also by lice as intermediate hosts [33]. Here, approximately 20% of dogs showed flea infestation at the time of examination. Although *A. reconditum* is less pathogenic than *D. immitis*, it is important to acknowledge the presence of this parasite, as it is a differential diagnosis for *D. immitis* if microfilariae are observed in blood samples.

Almost 60% of the *Ehrlichia*-seropositive dogs were currently infected (as defined as DNA detection by PCR in blood samples) with *E. canis*, i.e. at least 36.7% of all 329 dogs. In addition to *E. canis*, *E. ewingii* and *E. chaffeensis* also possess high zoonotic potential and antibodies against these *Ehrlichia* spp. may be detected by the used commercial rapid ELISA. However, we did not test for the presence of *E. ewingii* or *E. chaffeensis* by PCR here, as these pathogens have not been detected in ticks or domestic animals in Central America so far [10, 34, 35]. However, *E. chaffeensis* DNA was isolated from human patients in neighboring Costa Rica [36]. The principal vector of *E. ewingii* and *E. chaffeensis*, *Amblyomma americanum*, has not been reported to occur in Central America [37–39]. Nevertheless, it cannot be entirely excluded that *E. ewingii* and *E. chaffeensis* are present in Nicaragua; this should be considered for future studies as well.

Regarding *Anaplasma* spp., at least 6.6% of all dogs in the present study were currently infected. Both *A. platys* (*n* = 20), the causative agent of canine cyclic thrombocytopenia, and *A. phagocytophilum* (*n* = 17), which causes granulocytic anaplasmosis, were detected. In the majority of cases (*n* = 15), both species were present as co-infections. This result was somewhat surprising. As mentioned above, ixodid ticks, which are known to transmit *A. phagocytophilum*, are rather rare in Central America. Previously, only *A. platys* was reported to infect dogs in Nicaragua [15] and Costa Rica [10]; however, *A. phagocytophilum* has been found at low prevalence in *Rhipicephalus* ticks collected from dogs in Costa Rica [35]. It remains to be proven whether or not ticks other than *Ixodes* spp. may be implicated in the transmission of *A. phagocytophilum*. Nevertheless, the presence of *A. phagocytophilum* in Central America is especially important regarding the zoonotic potential of these pathogens. *Anaplasma phagocytophilum* frequently causes human granulocytic anaplasmosis, while the zoonotic potential of *A. platys* is considered to be low [4].

It cannot be excluded that there were further currently infected dogs amongst the seronegative animals, as antibody titres may not be detectable during the first 16 to 35 days post-infection [29]. However, seronegative animals were not tested by PCR. Therefore, we included only animals tested for current infections with *E. canis* and both *Anaplasma* species when assessing the impact of infections on PCV. In the statistical model, only *E. canis* infection had a significant negative impact on PCV. Likewise, Gaunt et al. [29] detected a significantly lower PCV in *E. canis*-infected dogs, but not in *A. platys*-infected dogs, compared to a control group. However, they reported that co-infection with both pathogens resulted in more severe anaemia than *E. canis* infection alone [29]. Here, only four individuals were co-infected with *E. canis* and *A. platys*, showing on average lower PCV values than those single-infected with *E. canis*. However, this phenomenon was not observed in the group of animals infected with all three pathogens, possibly because animals within this group were in different stages of infection. In general, co-infections may alter and/or exacerbate the clinical presentation of disease [3]; however, infections may also often be asymptomatic [4]. Here, apart from pale mucous membranes, clinical symptoms potentially related to ehrlichiosis or anaplasmosis were only rarely observed, and anorexia, which was frequently reported, was not associated with infection status.

## Conclusions

This study provides the first large-scale assessment of canine vector-borne diseases in Nicaragua. Similar to other Central American countries, a high prevalence of *E. canis* was detected, while *D. immitis* infections were rare and *B. burgdorferi* (*s*.*l*.) infections were not detected. *Anaplasma phagocytophilum* infections were observed almost as frequently as *A. platys* infections. Thus, dogs may constitute a reservoir for *A. phagocytophilum* in Nicaragua, which should not be neglected in the light of the zoonotic potential of this pathogen. Veterinarians and public health officials in Nicaragua should recommend the use of repellents or acaricides to protect dogs from vector-borne diseases, thus reducing the reservoir for human infection as well as the dog owners’ exposure to the vectors.
